# 2113. Evaluation of Isavuconazole Activity against *Aspergillus fumigatus* Causing Invasive Infections Worldwide Using the New CLSI Clinical Breakpoints

**DOI:** 10.1093/ofid/ofad500.1736

**Published:** 2023-11-27

**Authors:** Cecilia G Carvalhaes, Paul Rhomberg, Abby L Klauer, Beth Hatch, Mariana Castanheira

**Affiliations:** JMI Laboratories, North Liberty, IA; JMI Laboratories, North Liberty, IA; JMI Laboratories, North Liberty, IA; JMI Laboratories, North Liberty, IA; JMI Laboratories, North Liberty, IA

## Abstract

**Background:**

Isavuconazole (ISC) was approved by the US FDA and is considered first-line therapy for the treatment of invasive aspergillosis (IA). Azole resistance in *Aspergillus fumigatus* (AFM) is a growing concern, mainly caused by mutations within *CYP51* genes. The activity of ISC and other azoles against AFM causing IA worldwide was evaluated by applying the new CLSI ISC clinical breakpoints (BP).

**Figure:**
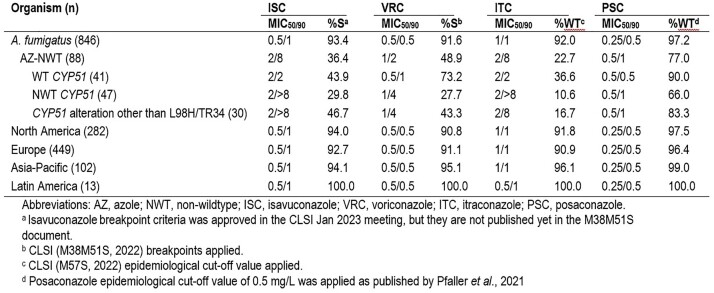

**Methods:**

A total of 846 AFM collected (1/patient) in 2017–2021 from 44 medical centers located in North America (NA; *n*=282; 18 centers), Europe (EU; *n*=449; 17 centers), Asia-Pacific (AP; *n*=102; 8 centers), and Latin America (LA, *n*=13; 1 center) were identified by MALDI-TOF MS and/or sequencing and tested by CLSI broth microdilution. CLSI BP and epidemiological cut-off values were applied where available. Isolates displaying a non-wildtype (NWT) phenotype to at least 1 azole were submitted to *CYP51* analysis by whole genome sequencing.

**Results:**

Overall, ISC (MIC_50/90_, 0.5/1 mg/L) showed similar activity to other azoles against AFM (Table), inhibiting 93.4% at ≤ 1 mg/L (CLSI-approved susceptible [S] BP). Voriconazole (VRC; MIC_50/90_, 0.5/0.5 mg/L), itraconazole (ITC; MIC_50/90_, 1/1 mg/L), and posaconazole (PSC; MIC_50/90_, 0.25/0.5 mg/L) inhibited 91.6%, 92.0%, and 97.2% at their respective S or wildtype criteria. The AZ-NWT phenotype was detected in 88 AFM (10.4%); NA showed the highest frequency of AZ-NWT isolates (31; 11.0%), followed by EU (48; 10.7%) and AP (9; 8.8%). No AZ-NWT AFM was noted in LA. Azole activity varied against AZ-NWT isolates with and without CYP51 alterations. Applying CLSI BPs, 43.9% of the AZ-NWT AFM without CYP51 alteration remained S to ISC and 73.2% were S to VRC. ISC and VRC inhibited 46.7% and 43.3%, respectively, of AZ-NWT AFM isolates displaying CYP51 alterations other than L98H/TR34 at the respective BP. AFM isolates carrying L98H/TR34 (17 occurrences) in the CYP51A sequence displayed elevated MIC ranges for all azoles: ISC, 2– > 8 mg/L; VRC, 1– > 8 mg/L; ITC, 2– > 8 mg/L; and PSC, 0.5–4 mg/L.

**Conclusion:**

ISC demonstrated potent *in vitro* activity against AFM regardless of the region and remained active against > 40% of AZ-NWT AFM isolates displaying wildtype CYP51 sequences or carrying CYP51 alterations other than L98H/TR34.

**Disclosures:**

**Cecilia G. Carvalhaes, MD, PhD**, AbbVie: Grant/Research Support|bioMerieux: Grant/Research Support|Cipla: Grant/Research Support|CorMedix: Grant/Research Support|Melinta: Grant/Research Support|Pfizer: Grant/Research Support **Paul Rhomberg, BS, MT(ASCP)**, bioMerieux: Grant/Research Support|Melinta: Grant/Research Support|Pfizer: Grant/Research Support **Abby L. Klauer, BS**, Pfizer: Grant/Research Support **Beth Hatch, BA MT(ASCP)**, Pfizer: Grant/Research Support **Mariana Castanheira, PhD**, AbbVie: Grant/Research Support|Basilea: Grant/Research Support|bioMerieux: Grant/Research Support|Cipla: Grant/Research Support|CorMedix: Grant/Research Support|Entasis: Grant/Research Support|Melinta: Grant/Research Support|Paratek: Grant/Research Support|Pfizer: Grant/Research Support|Shionogi: Grant/Research Support

